# Evaluation of sE-selectin and sPECAM-1 levels in patients diagnosed with food allergy

**DOI:** 10.1186/2045-7022-1-S1-P69

**Published:** 2011-08-12

**Authors:** Malgorzata Graczyk, Zbigniew Bartuzi, Michal Przybyszewski, Katarzyna Napiórkowska, Magdalena Zbikowska-Gotz, Jacek Muæka, Ewa Szynkiewicz, Robert Zacniewski, Ewa Socha

**Affiliations:** 1 Collegium Medicum in Bydgoszcz, Department of Allergology, Clinical Immunology and Internal Diseases, Bydgoszcz, Poland

## Introduction

PECAM-1 (CD31) (Platelet Endothelial Cell Adhesion Molecule-1) is an adhesive molecule found on the side surfaces of endothelial cells and blood platelets, taking part in the cell adhesion processes. Adhesive molecules are known to play an important part in the inflammatory processes, including inflammations with allergic background. The goal of the study was to assess the sE-selectin and sPECAM-1 levels in patients diagnosed with food allergies.

## Materials and Methods

80 patients were enrolled in the study, including 50 patients with food allergy and accompanying stomach pains, and 30 patients with dyspeptic discomfort without underlying food allergy. All subjects were subjected to gastroscopic examination, gastric mucosa biopsy for histopathological evaluation, including the presence of eosinophils in the inflammatory infiltrates, and assessment of Helicobacter pylori colonization status. Blood was collected from each subject for determination of the serum levels of the E-selectin and PECAM-1 adhesive molecules. sPECAM-1 and sE-selectin determinations were performed in a Bender MedSystems ELISA assay.

## Results

The average sE-selectin levels in the food allergy patient population were 54.0 +/- 21.6 ng/mL, while in the allergy-free population, the average sE-selectin levels were 57.7 +/-17.9 ng/mL. No statistically significant difference between sE-selectin levels was found between food allergy patients and patients with dyspeptic symptoms without concomitant food allergy (Mann-Whitney U-test, p =0.453028). In the food allergy patient population, average sPECAM-1 levels were 132.8 +/-31.4 ng/ml, while in the allergy-free population average sPECAM-1 levels were 139.6 +/- 31.1 ng/ml. The analysis of the obtained results revealed no statistically significant difference between sPECAM-1 levels in food allergy patients and patients with dyspeptic symptoms without concomitant food allergy.

## Conclusions

The results of examinations conducted in this study showed no statistically significant differences in serum PECAM-1 and E-selecine levels between the study groups.

